# Pharmacokinetic study of Q808 in rhesus monkey using liquid chromatography–tandem mass spectrometry

**DOI:** 10.3389/fphar.2024.1433043

**Published:** 2024-07-10

**Authors:** Ning Xiao, Xiang Li, Wei Li, Jialin Zhao, Yingnan Li, Limei Wang

**Affiliations:** ^1^ Office of Clinical Trial Institutions, Jilin Province FAW General Hospital, Changchun, China; ^2^ Department of Pharmacology, College of Basic Medical Sciences, Jilin University, Changchun, China; ^3^ Jilin Provincial Academy of Traditional Chinese Medicine, Changchun, China; ^4^ Department of Pharmacy, Jilin Province FAW General Hospital, Changchun, China; ^5^ Hand and Foot Surgery and Burn and Plastic Surgery, Jilin Province FAW General Hospital, Changchun, China

**Keywords:** epilepsy, Q808, LC-MS/MS, pharmacokinetic, rhesus monkeys

## Abstract

**Background:**

Q808 is a novel antiepileptic agent currently in development. In this study, we established and validated a LC-MS/MS method for the quantification of Q808 in Rhesus monkey plasma. Furthermore, we applied this method to investigate the pharmacokinetics of Q808 in Rhesus monkeys.

**Methods:**

Samples containing diazepam as an internal standard (IS) were subjected to liquid-liquid extraction (LLE) and separated using a Zorbax Extend C18 column. The detection of Q808 and IS was performed using multiple reaction monitoring mode (MRM), specifically monitoring precursor-to-product ion transitions at m/z 297.9 to 213.9 and m/z 285.2 to 193.1 for Q808 and IS, respectively. For the pharmacokinetic study of Q808, a total of 30 healthy Rhesus monkeys (half male and half female) were administered single oral doses, single IV doses, or multiple oral doses of Q808. Blood samples were collected at predetermined time points for subsequent pharmacokinetic analysis.

**Results:**

The developed LC-MS/MS method exhibited linearity within the concentration range of 1.5–750 ng/mL with intra-day precision ≤8.3% and inter-day precision ≤14.6%. Additionally, accuracy was found to be ≤ 3.4%. In the pharmacokinetic study involving single oral doses of Q808 in Rhesus monkeys, Q808 was absorbed with a median time to peak plasma concentration ranging from 4.50–6.00 h and was eliminated with a terminal elimination half-life (t_1/2_) between 9.34–11.31 h. No definitive conclusion regarding linear pharmacokinetic characteristics could be drawn. The absolute bioavailability was determined as 20.95%, indicating limited systemic exposure after oral administration. Multiple dosing did not result in significant accumulation based on an accumulation factor R_ac_ value of 1.31.

**Conclusion:**

We have successfully developed and validated a rapid yet sensitive LC-MS/MS method for quantifying levels of Q808 in rhesus monkey plasma for the first time. The determination method and pharmacokinetic characteristics of Q808 in rhesus monkey support the next steps in drug development.

## Introduction

Generalized convulsive status epilepticus (SE) is a life-threatening condition that poses a significant risk to individuals. Epilepsy is a prevalent neurological disorder affecting over 60 million people worldwide, across all age groups (Gavvala and Schuele, 2016). The annual incidence of epilepsy diagnosis is approximately 2%–3% (Gavvala and Schuele, 2016). Managing epilepsy often necessitates long-term medication and imposes substantial burdens on both individuals and society. Studies have shown that within 1 h, SE can lead to severe consequences such as hippocampal damage and the development of epilepsy (Lemos and Cavalheiro, 1995; Glien et al., 2001; Klitgaard et al., 2002; Rigoulot et al., 2004; François et al., 2006; Jung et al., 2006; Chu et al., 2008; Doeser et al., 2015; Löscher, 2015). Without appropriate antiepileptic treatments, the neurological sequelae of SE can be even more detrimental (Rossetti and Lowenstein, 2011; Löscher, 2015).

Recurrent unprovoked seizures and psychiatric symptoms like fear and anxiety are the primary manifestations of epilepsy, significantly impacting patients’ quality of life. The pathophysiology of epilepsy involves various mechanisms including neurotransmitter imbalance, channelopathies, neural migration abnormalities, neuronal loss, brain injuries, degenerative disorders, morphological abnormalities, cortical/hippocampal malformationsand genetic factors (Brodie et al., 2016; Pfisterer et al., 2020).

Long-term treatment with antiepileptic drugs (AEDs) is necessary for managing epilepsy. Although AEDs are widely used in treating SE to prevent further brain damage, they may cause adverse effects such as drowsiness, ataxia, gastrointestinal disturbances, hepatotoxicity, megaloblastic anemia (Leppik, 1994; Perucca, 1996; Lin and Kadaba, 1997; Sun et al., 2010) and potentially life-threatening conditions (Al-Soud et al., 2003; Sun et al., 2010). Additionally, a study has reported that AEDs were thought to trigger psychosis in up to 40% of cases (Thompson, 1999; Li et al., 2022b).

In recent years, tetrazoles have garnered attention as potential anti-seizure drugs due to their notable anti-inflammatory (El-Gazzar et al., 2008; Sun et al., 2010), antimicrobial (Karnik et al., 2006; El-Gazzar et al., 2008; Sun et al., 2010; Yu et al., 2012; Yu et al., 2014) anti-hypertensive (Israili, 2000; Sun et al., 2010) biological activities (Sun et al., 2010; Yu et al., 2014) as well as their ability to inhibit benzodiazepine receptor binding (Ambrogi et al., 1993; Sun et al., 2010). Therefore, the development of novel and safe agents with satisfactory efficacy and minimal side effects is imperative.

**FIGURE 1:**
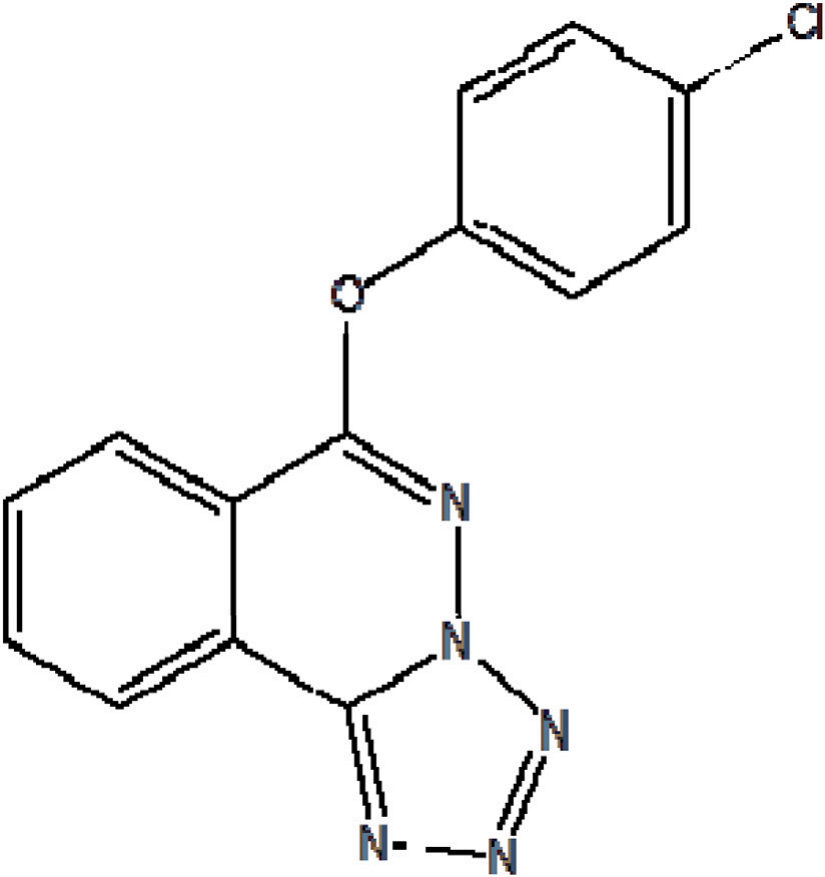
Chemical formulae of Q808.

An innovative chemical compound with an international patent, 6-(4-chlorophenoxy)-tetrazola [5, 1-a] phthalazine (Q808, Figure 1) is currently undergoing research and development. As previous studies reported (Li et al., 2017), Q808’s antiepileptic activity may be attributed to its ability to enhance r-aminobutyric acid (GABA) levels between synapses without affecting the function of GABA receptors. The underlying mechanism of Q808 involves modulation of the GABAergic system in specific brain regions, particularly increasing GABA levels in the hippocampus. Q808 has demonstrated effectiveness against various epilepsy models such as seizures induced by pentylenetetrazole (PTZ), isoniazid, thiosemicarbazide, and 3-mercaptopropionic acid (Li et al., 2022b). Previous studies have shown that a single dose of Q808 significantly increases GABA (Olsen, 1981; Okada et al., 1989; Li et al., 2017) in the rat hippocampal region while enhancing the frequency of spontaneous inhibitory postsynaptic currents in the hippocampus (Li et al., 2017; Li et al., 2022b). Treatment with Q808 also elevates angiotensin I-converting enzyme (ACE) levels—a protein related to GABA—potentially through upregulation of ACE gene and protein expressions in PTZ-kindled rats’ hippocampus (Li et al., 2022c).

Emerging studies suggest that gut microbiota reconstruction can positively impact epileptic seizures (Arulsamy et al., 2020; Li et al., 2022a). In this regard, Q808 can be utilized for remodeling dysbiosis within the gut microbiome while influencing neurotransmitter levels specifically within PTZ-induced seizure model rats’ hippocampus. Q808 treatment can also mitigate neuronal morphological changes and reduce the number of apoptotic cells in the hippocampus, while simultaneously decreasing epilepsy scores and prolonging stage 2 latency in rats (Cui et al., 2020; Li et al., 2024). Compared to Carbamazepine, the typical reference drug, Q808 exhibits superior anticonvulsant activity (ED_50_ = 6.8 mg/kg) against MES-induced seizures and lower neurotoxicity (TD_50_ = 456.4 mg/kg), as confirmed in mice (Perucca, 1996; Sun et al., 2010; Yu et al., 2014).

In this study, a LC-MS/MS method was developed and validated for the quantification of Q808 in Rhesus monkey plasma, which was successfully applied to investigate its pharmacokinetics in Rhesus monkeys. Based on reported ED_50_ and TD_50_ values, equivalent doses of Q808 for Rhesus monkeys were determined as 2.18 mg/kg and 146.32 mg/kg based on body surface area calculations. Therefore, dosages of Q808 administered in the Rhesus monkey pharmacokinetic study were selected as 6.44 mg/kg, 12.88 mg/kg, and 25.76 mg/kg within the range defined by ED_50_ and TD_50_.

## Materials and methods

### Chemicals and solvents

All chemicals and reagents from commercial sources were used as received unless stated otherwise. Q808 (purity 99.99%) was obtained from Jilin Provincial Academy of Traditional Chinese Medicine (Changchun, China). Diazepam (purity 99.9%) was purchased from the National Institute for the Control of Pharmaceutical and Biological Products (Beijing, China). Methanol (HPLC grade) and other solvents (analytical grade) were purchased from Concord Technology Company Ltd. (Tianjin, China). Milli Q water was prepared and used (Milli Q water systems, Millipore, Bedford, United States) throughout the study. Blank Rhesus monkey plasma (drug free and anticoagulated with heparin sodium) was prepared from Jilin Academy of Chinese Medicine Sciences.

### Instrumentation

The LC–MS/MS system consisted of an Agilent 1100 Series HPLC system (Agilent Technologies, Palo Alto, CA, United States) coupled to an Applied Biosystems Sciex Q-trap 2000 mass spectrometer (Applied Biosystems Sciex, Ontario, Canada) equipped with electrospray ionization (ESI) source. High purity nitrogen was used as the gas supply of the LC-MS/MS system. Data acquisition and integration were controlled by Analyst Software (Version 1.5.1, Applied Biosystems Sciex).

### LC-MS/MS conditions

The chromatographic separation was performed on a Zorbax Extend C18 column (15 cm × 4.6 mm I.D., 5 μm, Agilent, United States) maintained at 40°C using a mobile phase of methanol: 10 mM ammonium acetate 0.1% formic acid (85: 15, v/v). The flow rate was set at 1.0 mL/min and approximately 50% of the column eluent was introduced into the mass spectrometer through 1:1 split. The injection volume was 20 μL.

**FIGURE 2:**
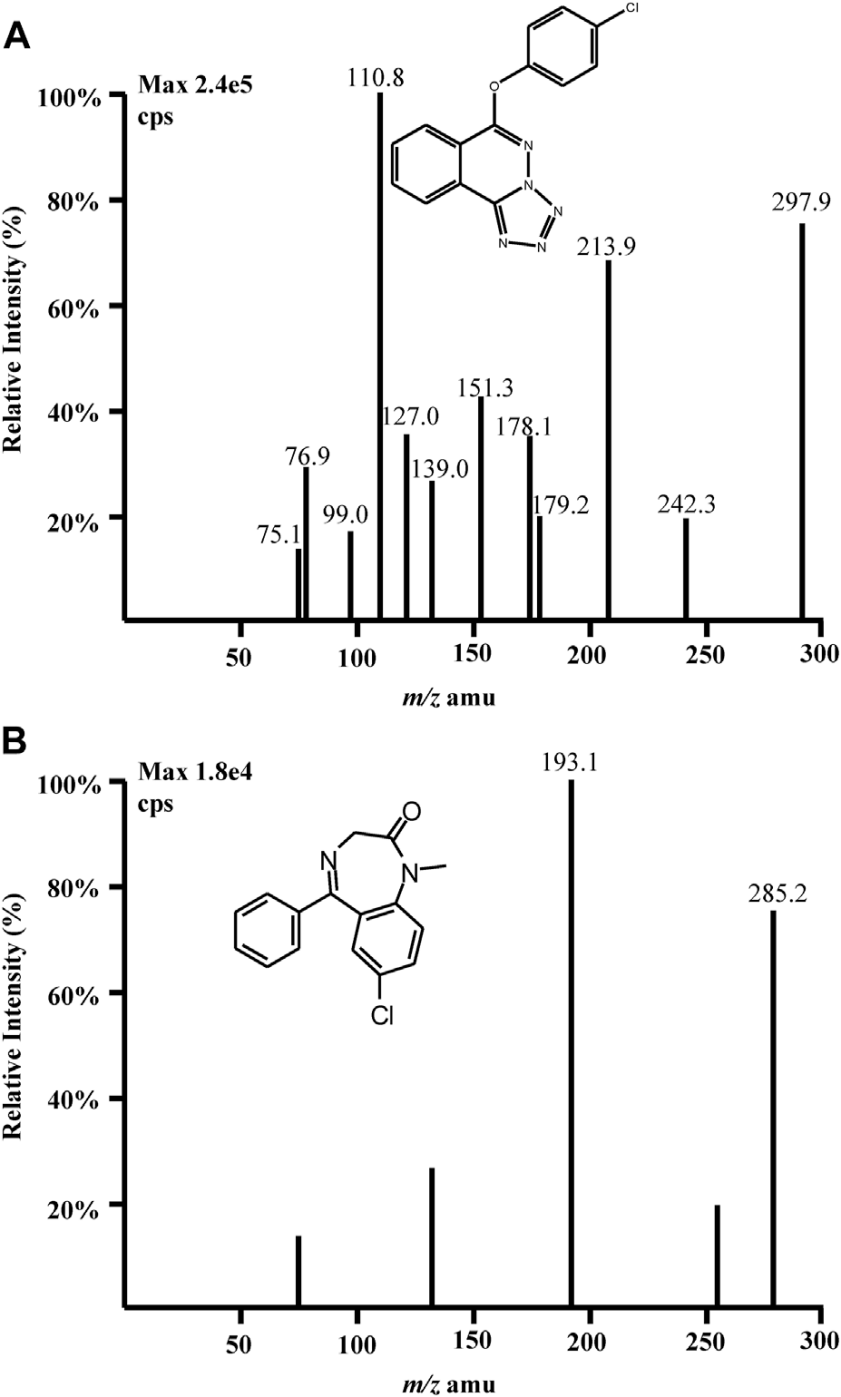
Full-scan product ion mass spectra of [M + H]+ ions of **(A)** Q808 and **(B)** diazepam in the positive ionization mode.

Quantitation was performed in MRM mode which was conducted by monitoring the precursor ion to product ion transitions for Q808 from m/z 297.9 (Q1) to m/z 213.9 (Q3) and m/z 297.9 (Q1) to m/z 110.8 (Q3) with the same declustering potential (DP) of 98 V and collision energies (CE) of 17 eV and 41 eV, respectively (Figure 2). The transition for diazepam (IS) was from m/z 285.2 (Q1) to m/z 193.1 (Q3) with DP of 80 V and CE of 42 eV. To optimize ESI conditions, Q808 and IS was dissolved in mobile phase and directly injected into the mass spectrometer. The optimized ionspray voltage was set at 5,000 V for positive ionization and source temperature was at 450°C. Nitrogen was used as nebulizing gas (35 psi), turbo gas (55 psi) and curtain gas (20 psi). The most abundant transition m/z 297.9→213.9 was chosen for the quantitation of Q808 and m/z 297.9→110.8 was used as the monitoring transition for confirming purposes. The pause time was set at 10 ms and dwell time was at 200 ms.

### Preparation of standard and quality control (QC) samples

A stock solution (1 mg/mL) of Q808 in methanol was diluted with methanol to obtain standard solutions at the concentrations of 30, 50, 150, 450, 1,500, 4,500, 15,000 ng/mL before use. Calibration standards were produced by spiking 1,900 μL aliquot of blank monkey plasma with 100 μL aliquot of the appropriate standard solutions to get the final concentrations of 1.5, 2.5, 7.5, 22.5, 75, 225, 750 ng/mL. Low, medium, high QC solutions (2, 20, 600 ng/mL) were obtained prepared in the same way. A stock solution (1 mg/mL) of IS in methanol was diluted with methanol: water (50: 50, v/v) to get an IS working solution with the final concentration of 150 ng/mL. All solutions were stored at 4°C and calibration standards and QC samples stored at −80°C before use.

### Sample preparation

Plasma samples were thawed at ambient temperature for 0.5 h before use. 50 μL aliquot of IS working solution, 50 μL aliquot of methanol and 200 μL aliquot of the plasma samples were added in a glass tube. The mixture was subjected to LLE using 3.5 mL hexane: dichloromethane: dimethyl carbinol (300: 150: 15, v/v/v). After the mixing, the mixture was vortex-mixed for 1 min and centrifuged at 3,500 rpm for 5 min. The upper organic layer was transferred to another tube and evaporated under gentle nitrogen at 40°C. The residue was reconstituted in 100 μL methanol: water (50: 50, v/v) and 20 μL was injected into the LC-MS/MS system for analysis.

### LC-MS/MS method validation procedure

The method was fully validated according to the Food and Drug Administration (FDA) guidance for biological method validation with respect to selectivity, linearity, lower limit of quantitation, accuracy and precision, recovery and matrix effects, dilution integrity, stability, carry-over and cross-talk effects.

Specificity was evaluated by analyzing drug-free monkey plasma samples collected from six rhesus monkeys with and without spiking with analyte and IS.

Intra- and inter-day precision (as relative standard deviation, RSD) and accuracy (as relative error, RE) were estimated by analyzing six replicate QC samples on three consecutive days and on the same day, respectively. The intra- and inter-day precision should not exceed 15%, the accuracy should be within 15%.

Linearity in the range from 1.5–750 ng/mL was evaluated by linear least-squares regression with a weighting index (1/*x* ^*2*^) of calibration curves based on peak area ratios prepared in duplicate from three separate batches. The lower limit of quantitation (LLOQ) was defined as the lowest drug concentration that could be determined with accuracy ±20% and precision <15% based on analysis of six replicate QC samples.

Recovery was assessed by comparing peak areas of analyte and IS in six replicates of QC samples at three concentrations with those of post-extracted blank plasma samples spiked at corresponding concentrations. Matrix effect was determined by comparing average peak areas of the analyte and IS in six replicates of dissolved with blank matrix extracts with those of corresponding solutions at the same concentrations.

Dilution integrity was evaluated by spiking the matrix with the analyte concentration above the upper limit of quantification (ULOQ) and diluting these samples with blank monkey plasma at five determinations at the dilution factors of 10 and 100. Accuracy and precision should be within ±15% and the dilution factors should cover the dilution factors employed in the test samples.

Stability of the analyte was investigated by analyzing three replications of QC samples under the following conditions: at room temperature (25°C) for 4 h (short-term stability); at −80°C for 30 days (long-term stability); and after three freeze-thaw cycles from −80°C to 25°C (freeze-thaw stability). Stability of processed samples was also evaluated by re-injecting the processed samples that have been stored for 6 h in the autosampler. Samples were considered stable if the concentration deviation was within 15% of that freshly prepared QC samples. Stability of Q808 and IS in stock solutions was assessed by comparing the solutions stored at 4°C for 7 days and 6 h at room temperature with freshly prepared stock solutions, respectively.

### Pharmacokinetic study

The pharmacokinetic study in rhesus monkeys comprised three parts: single oral dose, single IV bolus dose and multiple oral dose. All animal experimental procedures were carried out in accordance with the National Institutes of Health guidelines and were approved by the Institutional Ethics Committee for the Care and Use of Laboratory Animals of Jilin University. The monkeys were housed under standard conditions of temperature, humidity and light with food and water provided *ad libitum*. 18 healthy rhesus monkeys with half male and female were randomly divided into three dose cohorts (6 monkeys for each cohort) in single oral dose study. Q808 was orally administrated in 6.44 mg/kg, 12.88 mg/kg and 25.76 mg/kg after an overnight fast. Blood samples (2 mL) were collected into heparinized tubes at 0 (pre-dose), 1, 2, 3, 6, 9, 12, 15, 19, 24, 30, 36, 48, 60 and 72 h post-dosing in single oral dose study. For single IV bolus cohort, 6 healthy rhesus monkeys with half male and female were IV bolus administrated in 6.44 mg/kg. Blood samples were collected at 0, 1, 5, 10, 30, 90, 180, 360, 540, 720, 1,440 and 2,160 min. In multiple oral dose cohort, 6 healthy rhesus monkeys with half male and female were oral administrated 12.88 mg/kg once daily for 7 days. Blood samples were collected at 0 h on day 5 and day 6; 0, 1, 2, 3, 6, 9, 12, 15, 19, 24, 30, 36, 48, 60, 72, 84, 96, 108, 120, 132 and 144 h on day 7. Plasma samples were obtained by centrifugation at 15,000 × g for 10 min and stored at −80°C until analysis. Plasma pharmacokinetic data were analyzed by standard non-compartmental methods using WinNonlin version 7.0 (Certara United States Inc.) and the pharmacokinetic parameters included peak plasma concentration (C_max_), time to peak plasma concentration (T_max_), AUC from time 0 to the last timepoint with a quantifiable concentration (AUC_0-t_), AUC from time 0 to infinity (AUC_0-∞_), t_1/2_, clearance (CL/F), apparent volume of distribution (V_d_/F), mean residence time (MRT) and the parameters at steady state in multiple oral dose cohort: C_max,ss_, C_min,ss_, T_max,ss_, AUC_0-t,ss_, AUC_0−∞,ss_, CL/F_ss_, V_d_/F_ss_, and MRT_,ss_. The accumulation factor R_ac_ value was calculated by the ratio of the mean AUC_0-t,ss_ after multiple oral dose Q808 12.88 mg/kg to the mean AUC_0-t_ after single oral dose Q808 12.88 mg/kg, Linear mixed effects model was used to explore the relationship between dose and exposure. Linear pharmacokinetic conclusion can be drawn if the 95% confidence interval (CI) of the linear regression slope was completely contained within the decision interval [1+ln(0.8)/ln(8)∼1+ln(1.25)/ln(8)].

## Results

### Selectivity, linearity and LLOQ

**FIGURE 3:**
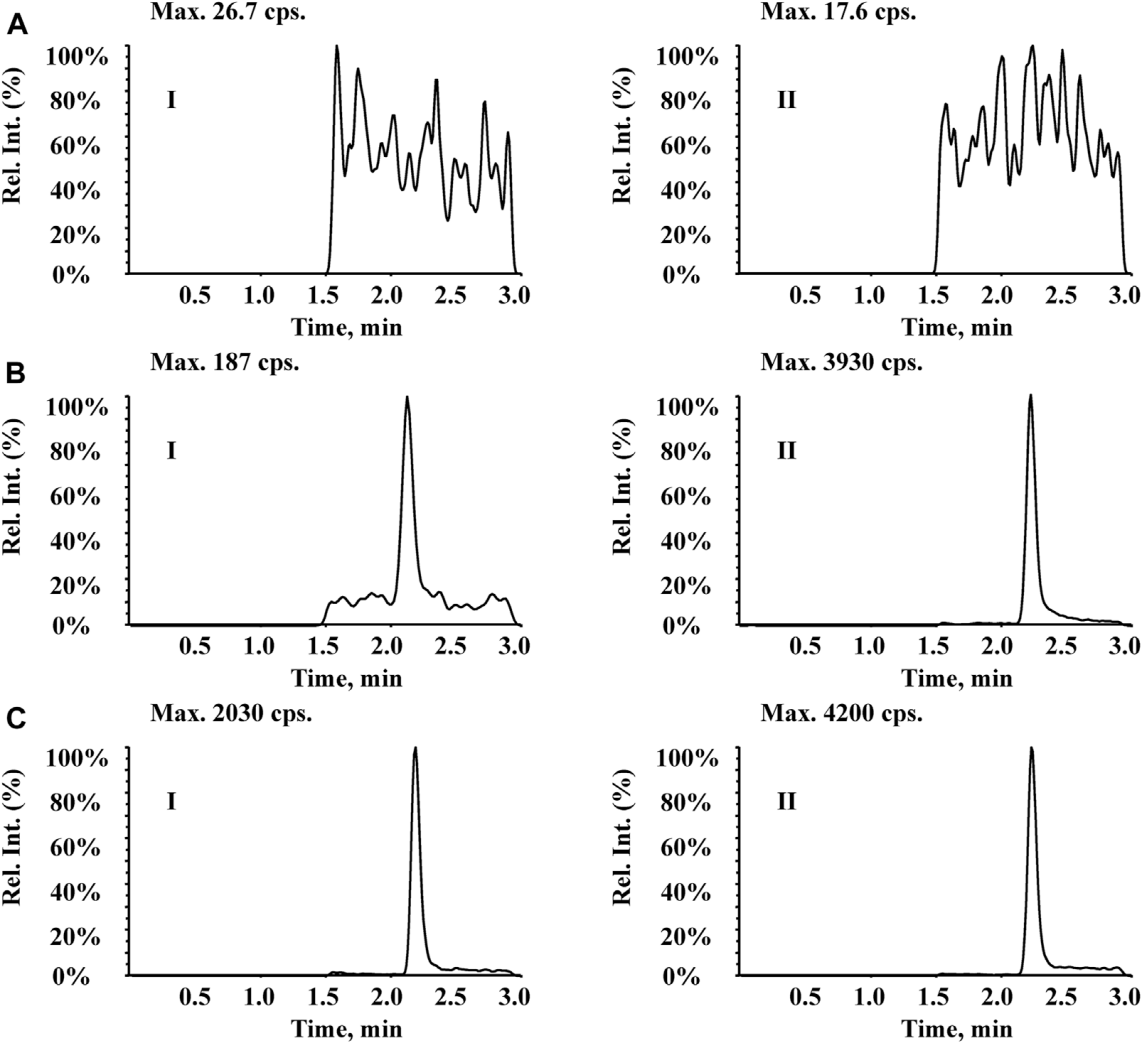
Typical MRM chromatograms for (I) Q808 and (II) IS diazepam in rhesus monkey plasma: **(A)** blank plasma; **(B)** blank plasma spiked with 1.5 ng/mL Q808; and **(C)** a plasma sample collected at 15 h after a high dose (12.88 mg/kg).

As shown in Figure 3A, no significant interference was observed from endogenous substances in plasma at the retention times of Q808 and diazepam. Figure 3B shows a representative ion chromatogram of a standard sample at a concentration of 1.5 ng/mL with a signal to noise ratio above 10. Figure 3C shows the chromatogram of a plasma sample collected at 15 h after a medium dose (12.88 mg/kg). The developed method was linear over the concentration range 1.5–750 ng/mL with correlation coefficients (R^2^) in the range of 0.9959–0.9978. A typical equation of a calibration curve was y = 0.00749x−0.00258, r = 0.9967. The LLOQ for Q808 was found to be identifiable, discrete, and reproducible with a precision of 20% and accuracy of 80%–120% (Table 1).

**TABLE 1:** Precision and accuracy of Q808 in rhesus monkey plasma (data was processed based on an assay of six replicates prepared QC samples on three different days, n = 24).

Spiked conc. (ng/mL)	Calculated conc. (ng/mL, mean)	Intra-day RSD (%)	Inter-day RSD (%)	Accuracy R.E. (%)
1.5	1.46	9.7	3.7	1.2
2	2.09	8.3	14.6	−0.4
20	21.7	5.5	8.5	−3.4
600	589	5.8	12.7	−1.7

### Precision and accuracy

Intra- and inter-day precision and accuracy data are presented in Table 1. Intra- and inter-day precisions (RSD) was ≤8.3% and 14.6%, respectively, with accuracy (RE) ≤3.4%.

### Extraction recovery and matrix effect

Extraction recoveries of Q808 for low, medium and high QC samples were at 102.53% ± 3.95%, 98.40% ± 3.46% and 90.01% ± 4.98%, respectively. Extraction recovery of IS was 92.5% ± 5.5%. The results indicated that recoveries of the analyte and IS were consistent and reproducible. The matrix effects for low, medium and high QC samples were 96.49% ± 5.31%, 99.91% ± 13.58% and 101.39% ± 3.20%, respectively, and they do not compromise the performance of the method at all.

### Dilution integrity

At dilution factors of 10 and 100, accuracy ranged from −7.50% to −12.0%, while precision was determined to be within a range of 1.98% to −13.0%. These values fell within acceptable limits for analytical standards (≤15%). A total of 37 samples were reanalyzed after being diluted due to their higher concentration exceeding the upper limit of quantification (750 ng/mL).

### Carry-over and cross-talk effects

Carry-over was estimated by injecting an extracted ULOQ sample and an extracted blank sample in turn, which is an extracted blank sample subsequently after an extracted ULOQ sample. Neither the analyte nor IS peak observed in blank samples after extracted ULOQ samples could indicate there was a slight carry-over after the ULOQ rejection.

The cross-talk was evaluated between the two SRM transitions used for monitoring the analyte and IS by injecting a processed matrix sample with IS and an extracted ULOQ sample respectively, and there was no cross-talk effect observed.

### Stability

The stability results demonstrate that Q808 and IS were stable under the conditions tested in Table 2 and in processed samples at room temperature for 6 h for the pharmacokinetic study.

**TABLE 2:** Stability of Q808 in Rhesus monkey plasma under various storage conditions (data was processed based on an assay of triplicate prepared QC samples at three concentrations, n = 3).

Storage conditions	Spiked conc. (ng/mL)	Calculated conc. (ng/mL)	R.E. (%)
		(mean ± SD, n = 3)	
In plasma at −80°C for 30 days	2.5	2.5 ± 0.2	−0.1
	22.5	20.1 ± 1.0	−10.7
	600	545 ± 9.9	−9.2
In plasma after three/thaw cycles	2.5	2.4 ± 0.3	−5.9
	22.5	22.1 ± 0.8	−1.8
	600	561 ± 7.0	−6.5
In plasma at room temperature for 4 h	2.5	2.6 ± 0.2	2.5
	22.5	24.3 ± 0.9	8
	600	586 ± 26.3	−2.2
In processed samples at room temperature for 6 h	2.5	2.5 ± 0.3	−2
	22.5	21.9 ± 2.9	−2.8
	600	652 ± 11.4	8.7

### Pharmacokinetic study

**FIGURE 4:**
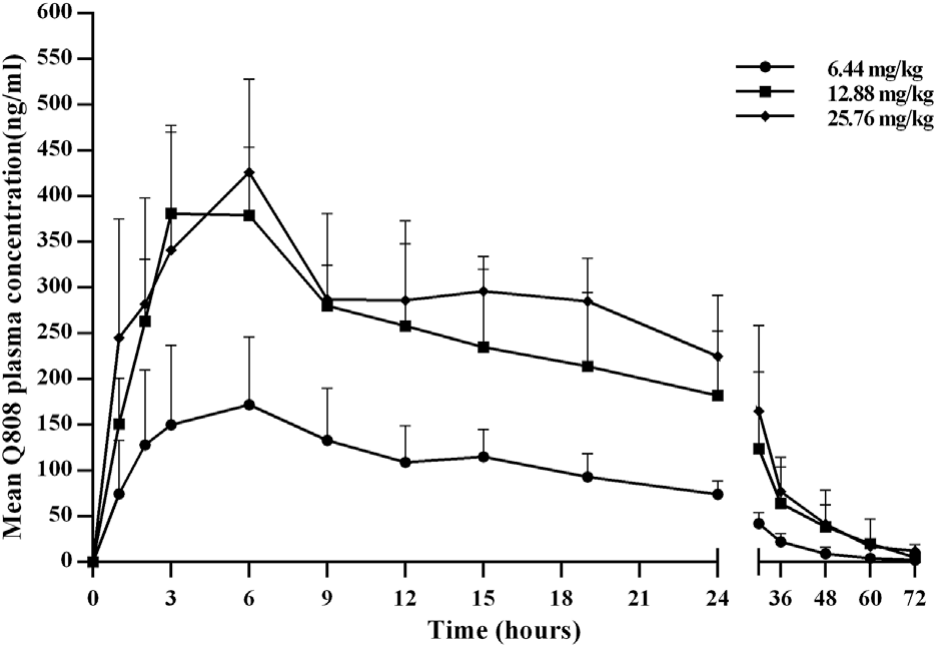
Mean plasma concentration-time profiles for Q808 after single dose 6.44 mg/kg, 12.88 mg/kg and 25.76 mg/kg Q808.

**FIGURE 5:**
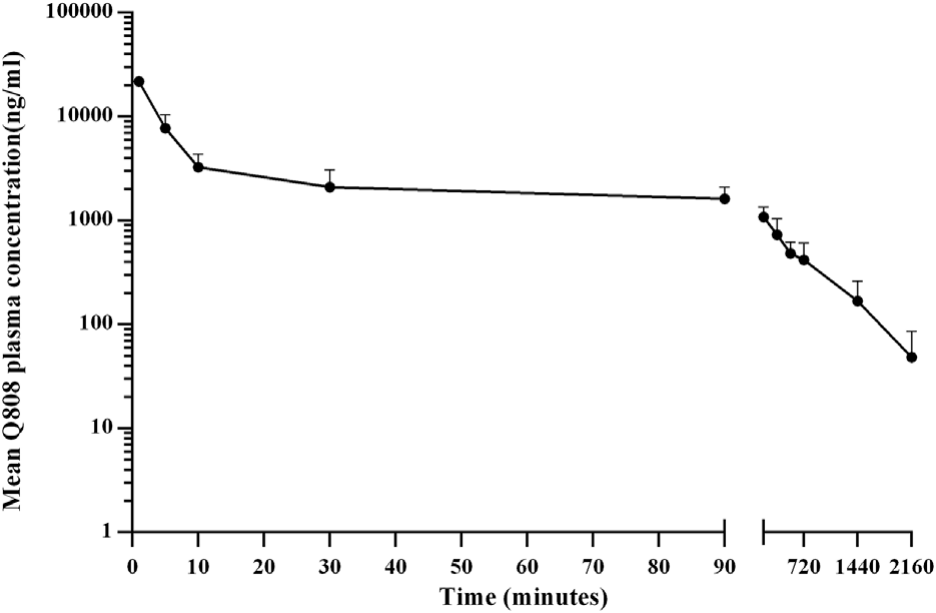
Mean plasma concentration-time profiles for Q808 after single IV dose 6.44 mg/kg Q808.

**FIGURE 6:**
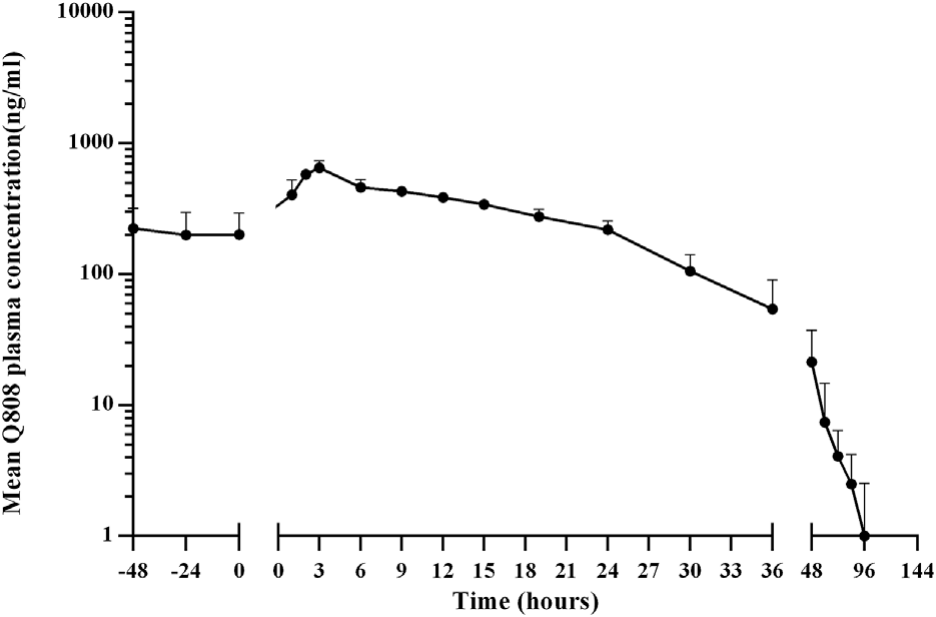
Mean plasma concentration-time profiles for Q808 after multiple dose 12.88 mg/kg Q808.

The validated analytical method was successfully applied to a pharmacokinetic study of Q808 in Rhesus monkeys after oral administration of Q808. The mean Q808 plasma concentration-time profile after single dose of oral administration are shown in Figure 4. The corresponding pharmacokinetic parameters were summarized in Table 3. After single dose of oral administration, Q808 was absorbed slowly with the median T_max_ 4.50–6.00 h, which is independent of dose. The mean t_1/2_ were 9.34–11.31 h and didn't show a proportional increase with increasing dosages of administration. The mean V_d_/F were 20,751.00–40109.33 mL/kg, larger than the total volume of plasma, indicating that Q808 distributes into tissues widely. The correlative analysis of dosage and pharmacokinetic properties were analyzed using WinNonlin linear mixed effects model and the slopes (95% CI) were 0.733 (0.44–1.02) and 0.774 (0.50–1.05) for ln C_max_ and ln AUC_0-t_, respectively, showing some overlap but not completely contained in the decision interval (0.84–1.16). Accordingly, no clear linear pharmacokinetic characteristic was observed. The mean Q808 plasma concentration-time profile after single IV bolus dose of Q808 are shown in Figure 5. The t_1/2_ was 7.58 ± 2.20 h in single IV bolus dose cohort, slightly shorter than oral administration. The absolute bioavailability was 20.95%, calculating using the ratio of AUC_0-t_ after single oral dose and single IV bolus Q808 6.44 mg/kg. Table 4 summarizes the plasma PK parameters after single IV bolus dose Q808 6.44 mg/kg. Figure 6 shows the mean Q808 plasma concentration-time profile after multiple oral dose Q808 12.88 mg/kg. In multiple oral dose cohort, visual inspection of Q808 trough concentration data indicated that steady-state were reached after approximately 5 days continuous administration. The accumulation factor R_ac_ value (calculating by AUC_0-t,ss_ after multiple oral dose Q808 12.88 mg/kg/AUC_0-t_ after single oral dose Q808 12.88 mg/kg) was 1.31, showing no obvious accumulation. Table 5 summarizes the plasma PK parameters after multiple oral dose Q808 12.88 mg/kg.

**TABLE 3:** Pharmacokinetic (PK) properties of Q808 after single oral administration of 6.44, 12.88 and 25.76 mg/kg.

PK parameter	Dosage (mg)			ANOVA (*p* Value)
	6.44 mg/kg	12.88 mg/kg	25.76 mg/kg	
No.	6	6	6	
T_max_, median (min–max), h	6.00 (3.00–9.00)	4.50 (3.00–12.00)	4.50 (1.00–12.00)	0.9092
C_max_, mean (SD), ng/mL	182.17 (81.81)	406.33 (75.46)	470.67 (77.39)	<0.0001
AUC_0-t_, mean (SD), h*ng/mL	3,589.17 (1028.66)	8,754.83 (2887.94)	10,225.00 (1384.63)	0.0007
AUC_0−∞_, mean (SD), h*ng/mL	3,648.33 (1027.93)	8,855.83 (2940.86)	10,444.00 (1480.45)	0.0006
T_1/2_, mean (SD), h	10.04 (2.592)	9.34 (2.85)	11.31 (2.86)	0.4769
V_d_/F, mean (SD), mL	27,650.17 (11690.63)	20,752.00 (8171.39)	40,109.33 (7377.86)	0.0312
CL/F, mean (SD), mL/h/kg	1,886.83 (538.60)	1,587.50 (488.57)	2,517.00 (429.35)	0.0099
MRT, mean (SD), h	17.92 (3.18)	18.48 (4.67)	20.22 (3.27)	0.5581

Abbreviations: ANOVA, analysis of variance; SD, standard deviation; T_max_, time to peak plasma concentration; C_max_, peak plasma concentration; AUC, area under the plasma concentration; T_1/2_, terminal elimination halt-life; V_d_/F, apparent volume of distribution; CL/F, apparent clearance; MRT, mean residence time.

**TABLE 4:** Pharmacokinetic (PK) properties of Q808 after single IV bolus of 6.44 mg/kg.

PK parameter	Dosage (mg)	t-test vs. 6.44 mg/kg single oral dose (p-Value)
	6.44 mg/kg	
No.	6	
T_max_, median (min–max), min	1.00 (1.00–1.00)	0.0022
C_max_, mean (SD), ng/mL	21,816.67 (658.53)	<0.0001
AUC_0-t_, mean (SD), h*ng/mL	17,131.50 (5,225.89)	0.0012
AUC_0−∞_, mean (SD), h*ng/mL	17,758.83 (5,492.25)	0.0013
T_1/2_, mean (SD), h	7.58 (2.20)	0.1127
V_d_, mean (SD), mL	4,129.67 (1128.37)	0.0042
CL, mean (SD), mL/h/kg	394.50 (127.59)	0.0008
MRT, mean (SD), h	8.90 (2.87)	0.0004

Abbreviations: SD, standard deviation; T_max_, time to peak plasma concentration; C_max_, peak plasma concentration; AUC, area under the plasma concentration; T_1/2_, terminal elimination halt-life; V_d_, apparent volume of distribution; CL, apparent clearance; MRT, mean residence time.

**TABLE 5:** Pharmacokinetic (PK) properties of Q808 after multiple oral administration of 12.88 mg/kg.

PK parameter	Dosage (mg)	t-test vs. 12.88 mg/kg single oral dose (p-Value)
	12.88 mg/kg	
No.	6	
T_max,ss_, median (min–max), min	2.67 (2.00–3.00)	0.0758
C_max,ss_, mean (SD), ng/mL	654.67 (85.99)	0.0022
AUC_0-t,ss_, mean (SD), h*ng/mL	11,467.83 (1,761.08)	0.0778
AUC_0−∞,ss_, mean (SD), h*ng/mL	11,509.50 (1,764.86)	0.0873
T_1/2,ss_, mean (SD), h	13.93 (7.36)	0.1850
V_d_/F_,ss_, mean (SD), mL	45,027.00 (24,536.95)	0.0260
CL/F_,ss_, mean (SD), mL/h/kg	2,278.33 (316.93)	0.0157
MRT_,ss_, mean (SD), h	15.50 (1.72)	0.1898
R_ac_	1.31	

Abbreviations: SS, steady state; SD, standard deviation; T_max_, time to peak plasma concentration; C_max_, peak plasma concentration; AUC, area under the plasma concentration; T_1/2_, terminal elimination halt-life; V_d_/F, apparent volume of distribution; CL/F, apparent clearance; MRT, mean residence time; R_ac_, AUC_0-t_ (multiple oral administration 12.88 mg/kg)/AUC_0-t_ (single oral administration 12.88 mg/kg).

## Discussion

A rapid and sensitive LC-MS/MS method was developed and validated for the determination of Q808 in rhesus monkey plasma for the first time. The method demonstrated adequacy, reliability, and was subsequently applied to the pharmacokinetic study of Q808 in rhesus monkeys.

For the development and optimization of the LC-MS/MS method, two ionization sources were considered: electrospray ionization (ESI) and atmospheric chemical ionization (APCI). However, due to its structure and relatively high polarity, APCI did not yield satisfactory signals for Q808. Consequently, mass spectrometry analysis was performed using an ESI source operated in positive ionization mode on a QTRAP 2,000 mass spectrometer (AB SCIEX, Toronto, Canada). During optimization of the mass spectrometric parameters, the most abundant quasi-molecular ions observed were m/z 297.9 for Q808 and m/z 285.2 for diazepam respectively. The product ion mass spectra of these protonated ions exhibited two major product ions at m/z 213.9 and m/z 110.8 for Q808 as well as one product ion at m/z 193.1 for diazepam. Optimization of chromatography conditions involved testing several reversed phase columns including Zorbax Extend-C18, Hypersil-C18, Venusil MP-C18 and Agilent TC-C18 along with various organic solvents and modifiers tested. Different ratios of organic solvents (methanol or acetonitrile) to water in mobile phase were evaluated. Higher ratios of acetonitrile in mobile phase (e.g., 70%, 80%, or 90%) resulted in analyte peaks approaching column frontiers; lower ratios of methanol in mobile phase (e.g., 50%, 60%, or 70%) prolonged analyte retention time. Medium range ratio combinations such as acetonitrile at approximately 50%–60% and methanol at higher percentages (80%–90%) yielded similar analyte retention times. Considering cost-effectiveness, methanol would be preferred. The absence of a termination in the Venusil MP-C18 column resulted in high background noise when injecting numerous biological samples. Under the same ratio condition of organic solvents, the retention time on the Hypersil-C18 column was longer. Considering all factors, the Zorbax Extend-C18 column proved to be the most suitable chromatographic column, with a mobile phase consisting of methanol and water (85:15, v/v).To optimize LC-MS/MS conditions, pH modifiers such as ammonium acetate, formic acid, and ammonia were included in the water phase. The addition of ammonia resulted in a decrease in the ionization efficiency and sensitivity of the analyte and IS, as well as low retention time. However, by incorporating formic acid into the aqueous component, peak shape improved significantly, while ammonium acetate provided a stable pH buffer system. Various concentrations of formic acid and ammonium acetate were tested to achieve symmetrical peaks, satisfactory sensitivity, and enhanced response. Consequently, the addition of 0.1% formic acid in 10 mM ammonium acetate yielded optimal results for separation and peak shapes. In terms of selecting an IS compound, several candidates including felodipine, diazepam, amber octahydroacridine, tramadol, metformin, acetaminophen, codeine fexofenadine diphenhydramine telmisartan sertraline were evaluated (Wang et al., 2005; Wang et al., 2004). Diazepam was chosen as the most suitable IS due to its structural similarity to Q808 along with satisfactory peak shape and good resolution from Q808 under the employed chromatographic conditions. Conversely, the retention times for other compounds were either too long or too short.

Several sample preparation methods, including protein precipitation, liquid-liquid extraction, and solid phase extraction, were considered and evaluated. PPT with methanol or acetonitrile resulted in significant matrix effects on the analyte and IS. Following the principle of similarity-intermiscibility theory, a mixture of hexane: dichloromethane: isopropanol (300:150:15, v/v/v) was used as the extraction solvent to maintain similar logP values between the solvent and analyte/IS. The LC-MS/MS method was employed for pharmacokinetic analysis in Rhesus monkeys following oral administration of Q808. After single administration, Q808 exhibited slightly slow absorption rates without dose dependence. The t_1/2_ values were comparable across all single dose cohorts and did not show proportional increases with higher dosages. The large V_d_/F value indicated wide distribution of Q808 into tissues, potentially facilitating its reach to target sites. No definitive conclusion regarding linear pharmacokinetic characteristics could be drawn within the dosage range of 6.44 mg/kg-25.76 mg/kg; however, correlative analysis revealed some linear trends. In the single oral dose cohort receiving a dosage level of 25.76 mg/kg, the increases of exposure and C_max_ were significantly lower than expected based on dose ratio calculations. This discrepancy is most likely attributed to absorption saturation observed in the high-dose group, potentially compounded by substantial inter-individual variation in PK parameters. The t_1/2_ after single IV bolus dose of Q808 was slightly shorter than after oral administration; multiple oral administrations showed a slightly longer t_1/2_ compared to single oral administration, but these differences were not considered significant due to individual variations among limited samples. No evident accumulation was observed after multiple oral administrations, supporting the safety of continuous medication.

## Data Availability

The original contributions presented in the study are included in the article/Supplementary Material, further inquiries can be directed to the corresponding author.
